# Kidney function and glucose metabolism in overweight and obese cats

**DOI:** 10.1080/01652176.2020.1759844

**Published:** 2020-05-02

**Authors:** L. Pérez-López, M. Boronat, C. Melián, Y. Brito-Casillas, A. M. Wägner

**Affiliations:** a Institute of Biomedical and Health Research (IUIBS), University of Las Palmas de Gran Canaria (ULPGC), Las Palmas, Spain; b Department of Endocrinology and Nutrition, Complejo Hospitalario Universitario Insular Materno-Infantil, Las Palmas de Gran Canaria, Spain; c Veterinary Faculty, Department of Animal Pathology, University of Las Palmas de Gran Canaria, Arucas, Las Palmas, Spain

**Keywords:** Cat, feline, obesity, metabolic syndrome, fructosamine, diabetes mellitus, nephropathy, symmetric dimethyl arginine, active transforming growth factor-β1, retinol biding protein

## Abstract

**Background:**

In people, obesity and prediabetes mellitus might predispose to chronic kidney disease (CKD).

**Aims:**

To assess the association of overweight [Body condition score (BCS) >5] and glucose metabolism alterations, with established or potential markers of CKD. In addition, fructosamine and fasted blood glucose were compared as predictors of early abnormal glucose metabolism.

**Methods:**

54 clinically healthy cats were included in a cross-sectional study comprising 25 neutered males and 29 (28 neutered) females aged 7.2 (5.5–9.4) years. Two potential markers of CKD, namely urinary free active transforming growth factor-β1-creatinine ratio and urinary retinol binding protein-creatinine ratio were measured along with other parameters to assess CKD. A receiver operating curve was used to identify the best sensitivity and specificity of fructosamine to identify cats with fasting glucose >6.5 mmol/L.

**Results:**

No association was found between BCS and markers of CKD. Fructosamine was greater in cats with fasting glucose >6.5 mmol/L compared to those with fasting glucose ≤6.5 mmol/L. A fructosamine concentration ≥250 µmol/L was able to detect cats with hyperglycemia with a sensitivity of 77% and a specificity of 65%. Furthermore, fructosamine was more strongly correlated with fasting glucose than albumin-corrected fructosamine (r = 0.43, p = 0.002 vs r = 0.32, p = 0.026). Cats with higher fructosamine had lower serum symmetric dimethylarginine concentrations.

**Conclusion:**

The present study does not suggest an effect of obesity on renal function in domestic cats.

**Clinical relevance:**

Fructosamine might be of value for the diagnosis of prediabetes mellitus in cats.

## 1. Introduction

In people, metabolic syndrome is defined as a cluster of disorders, which include abdominal obesity, abnormal glucose metabolism, dyslipidemia and hypertension (International Diabetes Federation, 2010; Matfin 2010). It is associated with a threefold risk of coronary heart disease and a fivefold risk of type 2 diabetes (T2DM) (International Diabetes Federation, 2010). Although T2DM is considered the leading cause of chronic kidney disease (CKD) in people (Pyram et al. 2012), recent studies have suggested that obesity, prediabetes and metabolic syndrome could also be independently associated with CKD (Johns et al. 2012; de Vries et al. 2014; Boronat et al. 2016; Markus et al. 2018). Feline diabetes mellitus is mostly classified as T2DM, with obesity being its main risk factor (Hoenig 2012; Nelson and Reusch 2014). Obesity can cause insulin resistance and dyslipidemia, and prediabetes has been suspected to occur in cats, too (Gilor et al. 2016), although diagnostic criteria are not well established. Fasting glucose levels may be considered impaired in cats when fasting glucose >6.5 mmol/L, whereas in the non-fasting state, it is considered that the upper normal limit is 9.2 mmol/L (Gottlieb et al. 2015; Reeve-Johnson et al. 2016; Gottlieb and Rand 2018). However, stress can increase blood glucose concentration in cats (Gottlieb and Rand 2018). In people, insulin resistance can be assessed through several simplified formulas that can be easily calculated from insulin concentration (Katz et al. 2000; Sung et al. 2010). In cats, fasting plasma insulin concentration and one of these formulas (the homeostasis model assessment, HOMA), have also been considered useful predictors of insulin sensitivity (Appleton et al. 2005), and could be potential markers of dysglycemia. However, measurement of insulin is expensive and requires fasting. Therefore, methods other than fasting glucose or insulin could be useful to study disorders of glucose metabolism. On the other hand, in contrast to people, the association of obesity and diabetes mellitus with CKD has not been fully investigated in cats. Thus, the present study was conducted in a sample of healthy cats with three different objectives: I) To assess the association between overweight (BCS >5) and a set of established or potential markers of kidney damage. II) To assess the association between different indicators of abnormal glucose metabolism and these same markers of kidney damage. III) To compare fructosamine and fasted blood glucose as predictors of early abnormal glucose metabolism.

## 2. Material and methods

### 2.1. Animals

A cross-sectional study was performed at the Veterinary Teaching Hospital of the University of Las Palmas de Gran Canaria. Clinically healthy cats, aged five years or above, were consecutively included. The owners participated voluntarily after signing an informed consent. Owners fulfilled a questionnaire covering information about previous diseases (including urinary tract disorders) and medical treatments. They were also specifically asked about clinical signs of diabetes mellitus or CKD, including unintentional weight loss, polyuria and polydipsia. Cats were considered healthy based on this questionnaire, a normal physical examination, normal abdominal ultrasound and blood tests. Exclusion criteria included previous diagnosis of a severe chronic disorder, such as lower urinary tract diseases, diabetes mellitus, CKD, leukemia or other neoplasms, owner-reported clinical signs of hyperglycemia or renal disease, positive test for retrovirus infections, use of corticosteroids or non-steroidal anti-inflammatory drugs in the previous six months, or treatment with other nephrotoxic drugs such as toceranib. CKD was defined as serum creatinine ≥140 µmol/L plus urinary specific gravity (USG) <1.035 or a urinary protein-creatinine ratio (UPC) >0.4 (International Renal Interest Society 2017). Those with serum creatinine concentrations ≥140 µmol/L, but not available urine data, were excluded as a diagnosis of renal failure could not be confirmed or ruled out. Cats were classified according to their body condition score (BCS; 1-9) as normal-weight (BCS = 5), as overweight (BCS = 6-7) or obese (BCS >7). Overweight and obese cats were combined into the overweight group (BCS >5) to simplify the interpretation of the results. All cats were assessed after a minimum of 12 hours of fasting (without water deprivation), and underwent physical examination, blood and urine sampling (cystocentesis or home collection), and an abdominal ultrasound. Systolic blood pressure was measured whenever possible, using a Doppler ultrasonic (Doppler Vet BP®, Mano Médical, Taden, France) or a high definition oscillometric (VetHDO^®^, S + B MedVet GmbH, Babenhausen, Germany) device, following international guidelines (Taylor et al. 2017).

This study was approved by the Animal Welfare Ethics Committee, University of Las Palmas de Gran Canaria, Spain with reference number 10/2018.

### 2.2. Analytical procedures

Blood samples were obtained in serum separator tubes, which were centrifuged and aliquoted within 20 minutes. Some of those aliquots were frozen. Creatinine, urea, glucose, alkaline phosphatase activity, alanine aminotransferase activity, total proteins, globulins, albumin and glucose were measured in fresh or refrigerated serum samples within 24 hours in all cats, whereas serum fructosamine, cholesterol, triglycerides and symmetric dimethylarginine (SDMA) were measured in refrigerated serum samples within 24 hours in 12 cats, and in frozen serum samples in 42 cats, all by spectrophotometry.

Insulin was measured with a commercial, feline insulin ELISA kit (Mercodia, Uppsala, Sweden). Insulin sensitivity was assessed through simplified estimation formulas [HOMA, quantitative insulin check index (QUICKI) and fasting insulin to glucose ratio (I/G)] (Appleton et al. 2005) (see Table 1).

**Table 1.** Simplified estimated formulas of insulin sensitivity.

Insulin sensitivity Index	Formula	
**HOMA**	(I_0_ x G_0_) / 22.5	
**QUICKI**	1/(log I_0_ + log G_0_)	
**Fasting I/G ratio**	I_0_ / G_0_	

I_0_ =fasting insulin *(μU/ml);* G_0_ = fasting glucose *(mmol/L)*

USG, urinary dipstick and urinary sediment examination were performed within 24 hours after urine collection. Those urine samples collected by the owner were preserved under refrigeration until the analysis was done; and those collected by cystocentesis were analyzed at the moment. Urine samples were aliquoted and frozen for later measurement of UPC by colorimetry (Animal Lab, Gran Canaria, Spain), and of the potential markers of renal disease: urinary free active transforming beta growth factor-creatinine ratio (uaTGFβ1:Cr) [Human Free Active TGF-β1 (BioLegend, San Diego, USA)] (Lawson et al. 2016) and retinol binding protein-creatinine ratio (uRBP:Cr) [human RBP sandwich ELISA kit (Immundiagnostik AG, Bensheim, Germany)] (van Hoek et al. 2008), were measured by commercial ELISA methods, following the manufacturers’ instructions. A standard curve was performed in each assay and all standards and samples were run in duplicate on the same plate. Absorbance was read at 450 nm within 30 minutes. The assay detection limits for Free Active TGF-β1 and RBP sandwich were 2.3 pg/ml (provided by the manufacturer) and 1.37 μg/l (provided by Hoek et al) (van Hoek et al. 2008), respectively.

### 2.3. Statistical analysis

Minimal sample size was calculated (Massachusetts General Hospital Biostatistics Center (MGHB) 2019) considering the standard deviation (0.41 µmol/L) and difference in means (0.50 µmol/L) in SDMA between cats with and without CKD, obtained from a previous study (Hall et al. 2014). A sample size of 16 cats in each group was required for a probability of 90 percent that the study would detect a difference of 0.50 µmol/L between groups of healthy cats and cats with CKD at a two-sided 0.05 significance level.

Distribution of quantitative variables was assessed through histograms. Data are presented as medians and interquartile ranges. Categorical variables are expressed as number of cats and percentages. Comparisons between groups were performed using the pairwise Mann–Whitney’s U test, and correlations between variables with the Spearman’s test. The comparisons and correlations were performed in two steps: first, to assess the associations between overweight or BCS and markers of kidney damage; and secondly, between markers of glucose metabolism and markers of kidney damage.

Albumin-corrected fructosamine was calculated according to the following formula (Reusch and Haberer 2001). fructosamine corrected for albumin (µmol/L) = observed fructosamine value (µmol/L) x median albumin concentration 31 (g/L)/observed albumin concentration (g/L).

A receiver operating curve (ROC) was used to find the fructosamine cut-off value with the best sensitivity and specificity for the detection of cats with fasting glucose >6.5 mmol/L (Gottlieb et al. 2015; Reeve-Johnson et al. 2016; Gottlieb and Rand 2018).

Statistical analysis was performed with SPSS Statistics Version 25.0 (IBM, Madrid, Spain).

## 3. Results

A total of 68 cats were examined for inclusion. Eight cats were excluded because CKD could neither be diagnosed nor ruled out, as their serum creatinine concentrations were ≥140 μmol/L and urine samples were not available. Two normal-weight cats were excluded because of CKD, and four additional cats (two normal-weight and two overweight) because of ultrasound findings consistent with urinary tract diseases (hydroureter (1), hydronephrosis (2), and bladder stones (1)). Thus, 54 clinically healthy cats were included, 25 neutered males and 29 (28 neutered) females, aged 7.2 (5.5-9.4) years. The breed distribution was as follows: domestic short hair (42), domestic long hair (4), mixed Persian (3), Persian (2), Siamese (2), and Angora (1). In total, 17 cats (6 male, 11 female) had normal BCS (BCS = 5), whereas 37 (19 male, 18 female) were overweight. Among these cats fructosamine could be measured in 52 cases, SDMA in 51 cats, uaTGFβ1:Cr in 29 cats, and uRBP:Cr in 28. Two cats had not fasted for 12 hours, so they were not included for the assessment of glucose, insulin or lipids. Therefore, for the assessment of glucose and lipid metabolism, 17 (6 male, 11 female) cats with BCS = 5, and 35 cats (17 males, 18 females) with BCS >5 were analyzed. Among them, fasting glucose was measured in 51 cats and fasting insulin was measured in 32 cats.

**Figure 1.**
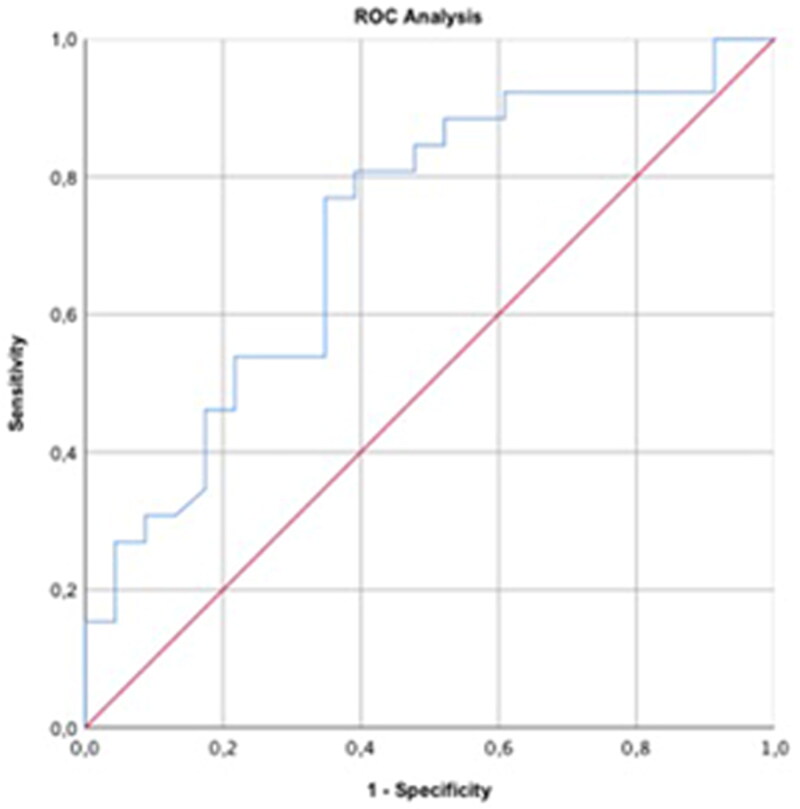
Sensitivity and specificity of fructosamine concentration to detect cats with a fasting glucose >6.5 mmol/L was calculated through ROC analysis (area under the curve = 0.72).

As expected, several variables reflecting abnormal glucose metabolism, including glucose, fructosamine, triglycerides, albumin and HOMA, were significantly greater in cats with BCS >5; and QUICKI was significantly lower in cats with BCS >5 (Table 2). Five out of 23 (21.7%) normal-weight cats, and 23 out of 26 (88.5%) cats with overweight had fasting serum glucose >6.5 mmol/L (p = 0.01). Fructosamine, but not albumin-corrected fructosamine, was significantly greater in cats with fasting glucose >6.5 mmol/L compared to those with fasting glucose ≤ 6.5 mmol/L (Table 3). In addition, fructosamine was more strongly correlated with fasting glucose than albumin-corrected fructosamine (r = 0.43, p = 0.002 vs r = 0.32, p = 0.026) and ROC analyses yielded a better diagnostic performance for fructosamine than for albumin-corrected fructosamine in the identification of cats with fasting glucose >6.5 mmol/L (area under the curve = 0.72 vs 0.61). Specifically, a fructosamine concentration ≥ 250 µmol/L was able to detect cats with a blood glucose concentration >6.5 mmol/L with a sensitivity of 77% and a specificity of 65% (Figure 1). Thus, albumin-corrected fructosamine was discarded for further analyses. Significant differences for fasting insulin and HOMA were observed when cats with a fructosamine ≥ 250 µmol/L were compared to cats with a fructosamine < 250 µmol/L (Table 4). Fructosamine was correlated with fasting insulin (r = 0.53; p = 0.002), HOMA (r = 0.56; p = 0.001), I/G ratio (r = 0.39; p = 0.028), and QUICKI (r = −0.56; p = 0.001).

**Table 2.** Clinical parameters assessed in 54 clinically healthy cats ≥5 years old, classified according to their body composition score (BCS). Data are given as median and IQR.

	Cats BCS = 5 (n = 17)	Cats BCS >5 (n = 37)	p-value*
Age *(years)*	7.0 (5.4–9.5)	7.3 (5.5–9.3)	0.963
**Weight *(kg)***	**3.8 (3.4**–**4.3)**	**5.4 (4.9**–**6.7)**	**<0.005**
**BCS *(1-9)***	**5.0 (5.0**–**5.0)**	**7.0 (6.0**–**8.0)**	**<0.005**
SDMA *(0-0.69 µmol/L)*	0.44 (0.32–0.64)	0.44 (0.39–0.54)	0.607
Creatinine *(71-212 µmol/L)*	132.6 (97.2–168.0)	150.3 (114.9–176.8)	0.123
Urea *(5.7-12.2 mmol/L)*	7.5 (6.5–8.7)	7.8 (6.8–9.0)	0.292
UsG (1.035-1060)	1050 (1044–1052)	1050 (1046–1055)	0.420
UPC (<0.4)	0.19 (0.09–0.43)	0.12 (0.09–0.19)	0.149
uaTGFβ1:Cr *(pg/mg)*	5.81 (2.70–8.40)	8.17 (4.63–12.65)	0.301
ALT activity *(12-130 U/L)*	42 (34–64)	50 (38–69)	0.280
ALKP activity *(14-111 U/L)*	35 (15–40)	27 (18–43)	0.730
**Albumin *(22-40 g/L)***	2.9 (2.8–3.2)	3.2 (3.0–3.4)	**0.001**
**Total protein *(57-89 g/L)***	7.0 (6.9–7.5)	7.6 (7.2–7.9)	**0.015**
Globulins *(28-51 g/L)*	4.1 (3.9–4.3)	4.3 (3.9–4.6)	0.308
**Fasting glucose *(4.1-8.8 mmol/L)***	**5.9 (5.1**–**8.7)**	**7.2 (6.3**–**9.3)**	**0.041**
**Fructosamine *(175-400 μmol/L)***	**246 (205**–**258)**	**257 (233**–**278)**	**0.046**
Corrected fructosamine *(175-400 µmol/L)*	249 (224–282)	251 (228–270)	0.733
**Triglycerides *(0.23-1.4 mmol/L)***	**0.48 (0.40**–**0.72)**	**0.90 (0.79**–**1.06)**	**<0.005**
Cholesterol *(2.6-10.6 mmol/L)*	3.78 (2.95–5.48)	4.40 (3.36–5.17)	0.321
Fasting insulin *(5.5-58.9 pmol/L)*	21.2 (15.3–29.3)	31.7 (18.4–80.6)	0.053
**HOMA**^+^	**0.75 (0.62**–**1.64)**	**1.77 (0.89**–**3.56)**	**0.040**
**QUICKI**^++^	**0.8 (0.6**–**0.8)**	**0.6 (0.5**–**0.8)**	**0.040**
Fasting I/G ratio^+^	0.58 (0.42–0.85)	0.84 (0.42–1.51)	0.158

Variables that showed a significant difference were highlighted in bold

ALT = alanine aminotransferase, ALKP = alkaline phosphatase BCS = body condition score, Fasting I/G = fasting insulin to glucose ratio, HOMA = homeostasis model assessment, QUICKI = quantitative insulin check index, SDMA = symmetric dimethylarginine, uaTGFβ1:Cr = urinary active transforming growth factor β: creatinine ratio, UPC = urine protein/creatinine ratio, USG = urinary specific gravity.

+ The higher the value, the lower the insulin sensitivity

++ The lower the value, the lower the insulin sensitivity

* p values <0.005 reflect a significant difference between cats with BCS = 5 and cats with BCS > 5

The uaTGFβ1:Cr was measured in 8 cats with BCS = 5, and 21 cats with BCS >5

The SDMA was measured in 17 cats with BCS = 5, and 34 cats with BCS >5

Fasting insulin was measured in 12 cats with BCS = 5 and 20 cats with BCS >5

Fasting glucose was measured in 17 cats with BCS = 5 and 34 cats with BCS >5

Fructosamine was measured in 17 cats with BCS = 5 and 35 cats with BCS >5

**Table 3.** Clinical parameters assessed in 51 clinically healthy cats ≥5 years old after 12 hours of fasting, classified according to their glucose concentrations. Data are given as median and IQR.

	Fasting glucose ≤6.5 mmol/L n = 23	Fasting glucose >6.5 mmol/L n = 28	p-value*
Age *(years)*	7.1 (5.5–9.6)	7.0 (5.5–9.4)	0.688
**Weight *(kg)***	**4.2 (3.8**–**5.2)**	**5.5 (4.5**–**6.8)**	**0.004**
**BCS *(1-9)***	**5.0 (5.0**–**6.0)**	**7.0 (6.0**–**8.0)**	**0.001**
SDMA *(0-0.69 µmol/L)*	0.44 (0.39–0.59)	0.39 (0.39–0.54)	0.356
Creatinine *(71-212 µmol/L)*	159.1 (88.4–176.8)	150.3 (132.6–168.0)	0.220
Urea *(5.7-12.2 mmol/L)*	7.7 (6.8–9.2)	7.5 (6.7–8.5)	0.550
UsG (1.035-1060)	1050 (1044–1053)	1050 (1047–1052)	0.645
UPC (<0.4)	0.17 (0.10–0.28)	0.13 (0.08–0.19)	0.147
uaTGFβ1:Cr *(pg/mg)*	6.45 (3.03–15.88)	7.71 (4.58–10.47)	0.983
**ALT activity *(12-130 U/L)***	**42 (35**–**57)**	**52 (42**–**74)**	**0.037**
ALKP activity *(14-111 U/L)*	33 (17–40)	29 (18–47)	0.715
**Albumin *(22-40 g/L)***	**2.8 (2.6**–**3.2)**	**3.2 (3.0**–**3.6)**	**0.033**
Total protein *(57-89 g/L)*	7.3 (7.0–7.8)	7.5 (6.9–7.8)	0.580
Globulins *(28-51 g/L)*	4.2 (3.9–4.6)	4.3 (3.9–4.5)	0.872
**Fasting glucose *(4.1-8.8 mmol/L)***	**5.8 (5.2**–**6.3)**	**8.3 (7.4**–**10.5)**	**<0.005**
**Fructosamine *(175-400 μmol/L)***	**235.2 (206.5**–**258.4)**	**261.4 (249.4**–**282.2)**	**0.007**
Corrected fructosamine *(175-400 µmol/L)*	242 (215–263)	254 (228–290)	0.207
**Triglycerides *(0.23-1.4 mmol/L)***	0.7 (0.4–0.9)	0.9 (0.7–1.1)	**0.009**
Cholesterol *(2.6-10.6 mmol/L)*	4.7 (3.0–5.4)	4.1 (3.4–4.4)	0.699
Fasting insulin *(5.5-58.9 pmol/L)*	20.7 (13.7–30.8)	33.9 (21.7–64.2)	0.073
**HOMA**^+^	**0.8 (0.5**–**1.4)**	**2.3 (1.7**–**4.5)**	**0.004**
**QUICKI**^++^	**0.8 (0.8-0.9)**	**0.6 (0.5-0.6)**	**0.001**
Fasting I/G ratio^+^	0.7 (0.4-0.9)	0.8 (0.4-1.4)	0.762

Variables that showed a significant difference were highlighted in bold

ALT = alanine aminotransferase, ALKP = alkaline phosphatase BCS = body condition score, Fasting I/G = fasting insulin to glucose ratio, HOMA = homeostasis model assessment, QUICKI = quantitative insulin check index, SDMA = symmetric dimethylarginine, uaTGFβ1:Cr = urinary active transforming growth factor β: creatinine ratio, UPC = urine protein/creatinine ratio, USG = urinary specific gravity.

+ The higher the value, the lower the insulin sensitivity

++ The lower the value, the lower the insulin sensitivity

* p values <0.05 reflect a significant difference between cats with fasting glucose ≤6.5 mmol/L and cats with fasting glucose >6.5 mmol/L

The uaTGFβ1:Cr was measured in 11 cats with fasting glucose ≤6.5 mmol/L, and 16 cats fasting glucose >6.5 mmol/L

The SDMA was measured in 22 cats with fasting glucose ≤6.5 mmol/L, and 26 cats with fasting glucose >6.5 mmol/L

Fasting insulin was measured in 17 cats with fasting glucose ≤6.5 mmol/L and 15 cats with fasting glucose >6.5 mmol/L

Fructosamine was measured in 23 cats with fasting glucose ≤6.5 mmol/L and 26 cats with fasting glucose >6.5 mmol/L.

**Table 4.** Clinical parameters assessed in 52 clinically healthy cats ≥5 years old after 12 hours of fasting, classified according to their fructosamine concentrations. Data are given as median and IQR.

	Cats Fructosamine <250 µmol/L N = 22	Cats Fructosamine ≥250 µmol/L N = 30	p-value
Age *(years)*	7.8 (6.0–9.7)	6.0 (5.4–9.3)	0.173
**Weight *(kg)***	**4.6 (3.8**–**5.3)**	**5.4 (4.3**–**6.7)**	**0.021**
**BCS *(1-9)***	**6.0 (5.0**–**7.0)**	**7.0 (5.5**–**8.0)**	**0.028**
**SDMA *(0-0.69 µmol/L)***	**0.54 (0.39**–**0.69)**	**0.39 (0.39**–**0.54)**	**0.021**
Creatinine *(71-212 µmol/L)*	159.1 (123.8–176.8)	150.3 (132.6–168.0)	0.707
Urea *(5.7-12.2 mmol/L)*	8.0 (7.2–9.2)	7.5 (6.5–9.0)	0.219
**UsG (1.035-1060)**	**1045 (1043**–**1051)**	**1050 (1048**–**1053)**	**0.032**
UPC (<0.4)	0.18 (0.10–0.26)	0.14 (0.09–0.25)	0.134
uaTGFβ1:Cr *(pg/mg)*	8.17 (5.21–11.35)	7.18 (3.56–13.41)	0.821
ALT activity *(12-130 U/L)*	46 (37–55)	56 (38–70)	0.210
ALKP activity *(14-111 U/L)*	33 (18–36)	33 (18–47)	0.470
**Albumin *(22-40 g/L)***	**3.0 (2.8**–**3.2)**	**3.2 (3.0**–**3.4)**	**0.029**
Total protein *(57-89g/L)*	7.2 (6.9–7.7)	7.5 (7.1–7.8)	0.219
Globulins *(28-51 g/L)*	4.2 (3.9–4.5)	4.3 (3.9–4.5)	0.930
**Fasting glucose *(4.1-8.8 mmol/L)***	**5.0 (5.9**–**6.7)**	**7.4 (6.5**–**9.2)**	**0.002**
**Fructosamine *(175-400 μmol/L)***	**219.9 (202.3**–**228.5)**	**285.1 (267.1**–**315.1)**	**<0.005**
Triglycerides *(0.23-1.4 mmol/L****)***	0.6 (0.5–1.0)	0.8 (0.6–1.0)	0.117
Cholesterol *(2.6-10.6 mmol/L)*	4.0 (3.0–5.1)	4.4 (3.5–5.0)	0.298
**Fasting insulin *(5.5-58.9 pmol/L****)*	**22.9 (13.0**–**31.5)**	**32.2 (20.9**–**63.4)**	**0.033**
**HOMA**^+^	**0.8 (0.6**–**1.7)**	**2.0 (1.1**–**4.0)**	**0.011**
**QUICKI**^++^	**0.8 (0.6**–**0.9)**	**0.6 (0.5**–**0.7)**	**0.017**
Fasting I/G ratio^+^	0.6 (0.4–0.9)	0.8 (0.5–1.7)	0.191

Variables that showed a significant difference were highlighted in bold.

ALT = alanine aminotransferase, ALKP = alkaline phosphatase BCS = body condition score, Fasting I/G = fasting insulin to glucose ratio, HOMA = homeostasis model assessment, QUICKI = quantitative insulin check index, SDMA = symmetric dimethylarginine, uaTGFβ1:Cr = urinary active transforming growth factor β: creatinine ratio, UPC = urine protein/creatinine ratio, USG = urinary specific gravity.

+ The higher the value, the lower the insulin sensitivity

++ The lower the value, the lower the insulin sensitivity

* p values <0.05 reflect a significant difference between cats with fructosamine concentration <250 µmol/L and cats with fructosamine concentration ≥250 µmol/L

The uaTGFβ1:Cr was measured in 13 cats with <250 µmol/L, and 15 cats with ≥250 µmol/L

The SDMA was measured in 21 cats with fructosamine <250 µmol/L, and 29 cats with fructosamine ≥250 µmol/L

Fasting insulin was measured in 16 cats with fructosamine <250 µmol/L and 16 cats with fructosamine ≥250 µmol/L

Fasting glucose was measured in 21 cats with fructosamine <250 µmol/L and 28 cats with fructosamine ≥250 µmol/L.

### 3.1. Association between overweight and markers of kidney damage

No statistically significant differences were found for established markers or potential markers of renal function between cats with BCS = 5 and cats with BCS >5 (see table 2). uRBP (not shown in the table) was measured in 28 cats (seven cats with BCS = 5, and 21 cats with BCS >5) but its concentrations were above the assay sensitivity in only eight cases (three with BCS = 5, and five with BCS >5); the median uRBP:Cr ratio was 0.81 × 10^−4^ (0.43 × 10^−4^–1.1 × 10^−4^) µg/mg, and no statistically significant differences were observed between groups (p = 1.0)

When assessed as continuous variable, no correlation was observed between BCS and established or potential markers of CKD [Creatinine (r = 0.019; p = 0.892); SDMA (r=-0.001; p = 0.994); USG (r = 0.136; p = 0.373); UPC (r=-0.102; p = 0.509), uaTGFβ1:Cr (r = 0.089; p = 0.645); uRBP:Cr (r=-0.048; p = 0.809)]. Systolic blood pressure was only measured in a total of 32 cats (8 cats with BCS = 5 and in 24 cats with BCS >5), and no statistically significant differences were observed [138 (116-144) vs. 137 (131-150) mmHg; p = 0.357].

### 3.2. Association between markers of glucose metabolism and kidney damage

Markers of kidney injury were compared between groups based on cut-offs for fasting glucose (6.5 mmol/L) and fructosamine (250 µmol/L) (see Table 3 and Table 4). There was no significant difference in any marker of kidney injury when cats were classified according to their glucose concentrations (see Table 3), whereas cats with a fructosamine <250 µmol/L showed higher SDMA than those with fructosamine ≥250 µmol/L (p = 0.021). In addition, moderate, inverse correlation was found between fructosamine and SDMA (r = −0.36; p = 0.011).

## 4. Discussion

As observed in previous studies (Appleton et al. 2001; Jordan et al. 2008; Hoenig 2012), we found a strong association between excess of body weight, insulin resistance and dyslipidemia, confirming the existence of a feline form of the metabolic syndrome. It is also known that cats with obesity are at greater risk of developing diabetes mellitus (Donoghue 1998; Hoenig 2012). However, the role of diabetes mellitus on feline CKD is unclear, and whether obese or prediabetic cats have a higher risk of CKD, has not been investigated yet. Since cats are frequently obese for most of their life, and they are chronically exposed to metabolic changes induced by obesity, particularly insulin resistance and the metabolic syndrome, we hypothesized that it might predispose them to develop CKD. Moreover, CKD is a prevalent disease in elderly cats (Reynolds and Lefebvre 2013), and most cases of CKD are of unknown cause, which could lead to the speculation that obesity could be a hidden causal factor for kidney injury. However, according to our results, concentrations of established markers of CKD, such as creatinine or SDMA, did not differ between normal-weight and overweight cats. In regard to the potential biomarkers assessed in this study, uaTGFβ has been proposed as an important mediator of diabetic nephropathy in animal models, one of which showed that urinary aTGFβ1:Cr ratio precedes the onset of azotemia by six months (Lawson et al. 2016). In relation to RBP, its urine concentration might increase when tubular damage occurs, and higher levels of urinary RBP:Cr in cats with CKD compared to healthy cats have been reported (van Hoek et al. 2008). However, these putative early markers of kidney injury were not correlated with obesity in our study. These findings add to previous studies (Greene et al. 2014; Freeman et al. 2016) suggesting that, in contrast to dogs (Henegar et al. 2001; Tvarijonaviciute et al. 2012; 2013), obesity does not contribute to the development of CKD in cats. ^.^ However, prospective studies would be required to definitely evaluate whether feline kidney function is affected by obesity.

On the other hand, our results suggest that changes in kidney function might exist related to higher blood glucose concentration. SDMA is a byproduct of arginine-methylated proteins, and is mainly excreted through the kidneys (Schwedhelm and Boger 2011). Its serum concentrations are inversely correlated with glomerular filtration rate (GFR) (Hall et al. 2014), and values persistently above 0.69 μmol/L are consistent with CKD (International Renal Interest Society 2017). One previous study reported that SDMA levels were lower in cats with diabetes mellitus than controls, but also compared to cats with CKD or cats with hypertrophic cardiomyopathy (Pyram et al. 2012)^5^. The authors suggested that it might be due to osmotic diuresis or hyperfiltration. The latter is one of the earliest changes observed in people with diabetic nephropathy (Dronavalli et al. 2008), and lower concentrations of SDMA due to hyperfiltration mechanisms have also been suggested in humans (Marcovecchio et al. 2010). In the present study, although neither cats with BCS >5 nor cats with fasting hyperglycemia showed reduced concentrations of SDMA, those with fructosamine concentration >250 µmol/L did have low SDMA concentrations. Osmotic diuresis could not explain low SDMA values in cats with higher concentrations of fructosamine, since the animals included in this study did not have glycosuria. Whether or not lower concentrations of SDMA are related to hyperfiltration requires further investigation.

Our study also assessed the possible role of serum concentrations of fructosamine for the evaluation of early abnormalities of glucose metabolism in cats. Recently, the use of blood glucose has been proposed to screen for prediabetes (Gottlieb et al. 2015; Reeve-Johnson et al. 2016; 2017; Gottlieb and Rand 2018). The authors proposed that cats with fasting glucose persistently >6.5 mmol/L could be considered prediabetic (Marcovecchio et al. 2010; Gottlieb et al. 2015; Gilor et al. 2016; Reeve-Johnson et al. 2016). However, repeated blood sampling and prolonged fasting are not easy to perform in clinical practice. In addition, blood glucose concentration is subject to the effect of stress, which could raise glucose levels up to 10.8 mmol/L (Gottlieb and Rand 2018). In the present study, high fructosamine concentrations showed a better association with markers of insulin resistance (fasting insulin and HOMA) than fasting glucose. As HOMA and fasting insulin have been considered reliable parameters to evaluate insulin sensitivity in cats (Appleton et al. 2005), this means that fructosamine could reflect decreased insulin sensitivity better than fasting glucose, maybe due to the fact that fructosamine is not affected by stress (Crenshaw et al. 1996). Fructosamine levels depend on blood glucose concentration and the half-lives of the proteins (Crenshaw et al. 1996). It has been assumed that feline fructosamine, as in dogs, could reflect blood glucose levels of the preceding one to two weeks (Dixon et al. 1953; Crenshaw et al. 1996). Furthermore, for fructosamine to exceed the normal reference range, severe hyperglycemia, lasting for 3-5 days is necessary (Link and Rand 2008). Therefore, it is considered a useful parameter to distinguish between diabetes mellitus and stress hyperglycemia (Crenshaw et al. 1996), and we hypothesized that it might be useful for the detection of feline prediabetes. In addition, fructosamine has some advantages compared to other methods: it is not time-consuming or expensive, nor does it require fasting. Prospective studies would be needed to evaluate this marker as a tool to diagnose prediabetes. In addition, it should also be highlighted that there is a lack of standardization in the methodology of fructosamine measurement among laboratories. Therefore, for a correct interpretation of fructosamine values, it should always be measured under the same methodology and laboratory (Sparkes et al. 2015; Idexx Reference Laboratories Support 2019).

Some limitations are acknowledged in this study. First, its cross-sectional character does not allow to establish causal inferences. Another important limitation is that the sample size was small and calculated to detect inter-groups defined differences in SDMA concentrations. Therefore, it might be underpowered to detect differences in other markers of kidney damage and its results should not be overestimated. Another limitation might be related to fasting. Cats were fasted for at least 12 hours; however, the postprandial period in cats could last longer, and it could affect concentrations of fasting blood glucose and fasting insulin (Appleton et al. 2001; Farrow et al. 2012).

In conclusion, the present study does not suggest an effect of obesity on renal function in domestic cats, whereas some changes in kidney function, reflected by SDMA concentrations, might be associated to mild chronic hyperglycemia. Finally, we propose fructosamine for the diagnosis of prediabetes mellitus, though optimal cut-offs should be investigated.
